# Dynamic Bargain Game Theory in the Internet of Things for Data Trustworthiness

**DOI:** 10.3390/s21227611

**Published:** 2021-11-16

**Authors:** Appasamy C. Sumathi, Muthuramalingam Akila, Rocío Pérez de Prado, Marcin Wozniak, Parameshachari Bidare Divakarachari

**Affiliations:** 1 Department of CSE, KPR Institute of Engineering and Technology, Coimbatore, Tamil Nadu 641407, India; sumathi.ac@kpriet.ac.in (A.C.S.); akila.m@kpriet.ac.in (M.A.); 2 Telecommunication Engineering Department, University of Jaén, 23071 Linares, Spain; 3 Faculty of Applied Mathematics, Silesian University of Technology, 44-100 Gliwice, Poland; 4 Department of Telecommunication Engineering, GSSS Institute of Engineering and Technology for Women, Mysuru 570016, India; paramesh@gsss.edu.in

**Keywords:** data trustworthiness, dynamic bargaining game, internet of things, smart homes, packet delivery ratio, wireless sensor networks

## Abstract

Smart home and smart building systems based on the Internet of Things (IoT) in smart cities currently suffer from security issues. In particular, data trustworthiness and efficiency are two major concerns in Internet of Things (IoT)-based Wireless Sensor Networks (WSN). Various approaches, such as routing methods, intrusion detection, and path selection, have been applied to improve the security and efficiency of real-time networks. Path selection and malicious node discovery provide better solutions in terms of security and efficiency. This study proposed the Dynamic Bargaining Game (DBG) method for node selection and data transfer, to increase the data trustworthiness and efficiency. The data trustworthiness and efficiency are considered in the Pareto optimal solution to select the node, and the bargaining method assigns the disagreement measure to the nodes to eliminate the malicious nodes from the node selection. The DBG method performs the search process in a distributed manner that helps to find an effective solution for the dynamic networks. In this study, the data trustworthiness was measured based on the node used for data transmission and throughput was measured to analyze the efficiency. An SF attack was simulated in the network and the packet delivery ratio was measured to test the resilience of the DBG and existing methods. The results of the packet delivery ratio showed that the DBG method has higher resilience than the existing methods in a dynamic network. Moreover, for 100 nodes, the DBG method has higher data trustworthiness of 98% and throughput of 398 Mbps, whereas the existing fuzzy cross entropy method has data trustworthiness of 94% and a throughput of 334 Mbps.

## 1. Introduction

Internet of Things (IoT) networks consist of many sensors and devices connected to the Internet for communication and data collection. IoT applications and services have gained popularity for various reasons such as flexibility and scalability, and are used in applications such as home appliances, industries, and smart cities. The smart home system provides the convenience of connecting household applications to a single network for control and management. Home automation systems involve devices for lighting, thermostats, air conditioning, lawn/gardening management, and smart door locks. Specifically, the smart home generally involves the application of various types of sensors, such as a thermal sensor (electronic thermistor sensor) for temperature monitoring, a camera sensor (CMOS sensor) for security, a humidity sensor for moisture detection, and a passive infrared (PIR) for motion sensor. Moreover, the home automation system requires the sensor devices to be connected to the cloud and are usually controlled from the user’s mobile. Furthermore, real-time IoT networks provide many benefits and also suffer from various security vulnerabilities, such as data leakage, multi-latency, side-channels, and cross-site scripting [1,2,3]. An IoT sensor network collects data from a source node and passes it to the multiple intermediate sensor nodes to reach a destination node. The Base Station (BS) in the IoT network allows the destination nodes to communicate to the gateway. Sensor data reliability and trustworthiness are important for the data in several critical decisions in a real-time IoT network [4,5]. The rapid development of the IoT in mobile applications increases the requirement of feasibility (stable transmission) of the underlying Wireless Sensor Networks (WSNs), considering factors such as data trustworthiness, low power consumption, ultra-low latency, and security [6]. In this context, node trustworthiness is fundamental to the development of IoT networks for decision processes based on observation. Although some security methods provide high data trustworthiness, they are difficult to apply in an IoT environment due to cost and performance reasons [7].

Globally accessible devices and resource-constrained interconnections via an unreliable and untrusted Internet are vulnerable to attacks using packet drops, false data injection, and data forging, which affect the decision-making processes in applications. The provenance reliance for data trustworthiness is considered an effective method to track data transmission and data acquisition [8,9]. Most traditional global detection methods used in building secure networks focus on the nodes encounter ratio and requires holistic cognition for the network structure. Real-time IoT applications with incomplete and large-scale structures have limitations, such as instability in dynamic networks and lower security [10,11,12]. IoT networks are affected by various attacks, such as Selective Forwarding (SF), eavesdropping, sniffing, Man-in-the-Middle, and Denial of Services (DoS). Cyber-attacks can be applied to the targeted network to steal data, thus causing significant disruption to IoT systems. Various methods in the IoT-WSN have been developed and applied to improve the security of networks [13]. Recent mitigation methods to improve security in IoT networks are trust-based approaches, Intrusion Detection Systems, and machine learning for routing and malicious node discovery [14,15]. A secure method with high data trustworthiness and low end-to-end delay is required to provide a flexible, reliable, and effective real-time home automation system.

Various types of models have been applied to improve the data trustworthiness of networks, and the commonly used types of models are discussed in the following. A node selection method based on neighborhood information [1] was applied to eliminate the malicious nodes in the network. The direct and indirect trust among nodes was measured to reduce the packet drop ratio and improve the packet data rate among different nodes. The Digital Twin (DT) method [2] was applied to find the faults in the network and then take the necessary precaution to prevent the network from failing. The fault diagnosis system helped to improve the efficiency of the network and increased the packet delivery ratio. A probabilistic graphical model [3] was used to measure the trust between the nodes in the network based on data collection and communication behavior. The developed method helped to improve the data trustworthiness in the network and improved the efficiency of the model.

The Reversible Watermarking and Asymmetric Cryptography (AC) method [4] has been used to improve the data trustworthiness and ensure integrity. The developed method has higher efficiency than Reversible Watermarking, while ensuring integrity and lowering performance in terms of security. An elastic slide window and machine learning method [5] was used to improve the data trustworthiness and increase the security of the network. The developed method has lower efficiency in terms of detecting malicious nodes and node selection. Trust measurement models [1,3] provide an effective measure for the detection of malicious nodes, but fail to select the proper node for transmission. The water marking [4], fault detection [2] and machine learning [5] methods have lower efficiency in malicious node detection.

In comparison, the trust value has been measured from multi-dimensional data [6], and the trust value was mapped to find the node. Direct trust and indirect trust were measured from the network, and the best node was found for transmission. An assurance policy template [7] was applied for the trust measure based on the data collection process and human behavior. The assurance policy instance is applied in the assurance policy template for the selection of node. The efficiency and adaptability of the model is low in the network. The routing protocol method [8] was used for low-power and lossy networks to identify malicious nodes or faults in the nodes. The collected data was analyzed to study the malicious node and improved the efficiency of the model. A Markov Decision Process (MDP) [9] was applied for allocation of resources to service and encode a service provisioning system. Reinforcement Learning (RL) was applied to find the node to improve the efficiency. The trained policy increased the trustworthiness in the model.

The Detection Scheme for Dynamic Trustworthiness Overlapping Community (D2-TOC) was applied to improve data trustworthiness [10]. The node pair information, such as service degree, recency and contact probability, were measured for data trustworthiness. The developed method had lower performance in terms of the efficiency of the network. The trust-based methods [6,7] showed a strong performance in node selection and a lower performance in malicious node detection. The routing protocol [8] had higher performance in detecting malicious node and lower efficiency in the dynamic network. The reinforcement learning and MDP [9] provided higher efficiency network allocation, which failed to operate in the dynamic network. The overlapping-based model [10] had lower efficiency in node selection for transmitting the data.

The Analytical Network Process (ANP) [11] was applied to improve data trustworthiness and efficiency. The pairwise comparison was applied to analyze the criteria and available alternatives. The Supervisory Control and Data Acquisition (SCADA) method [12] was applied for a reliable and scalable network in a cyber-attack detection model. The Random Subspace (RS) with the Random Tree (RT) was applied for detection of cyber-attacks. The ANP [11] method had lower efficiency in the detection of the malicious node and Random Tree [12] method had an overfitting problem in the detection.

Hence, the existing methods used in IoT security have the limitations of low data trustworthiness, low adaptability, and high latency. The existing methods used in path selection to improve the IoT-WSN security have the limitations of lower performance in the dynamic network and low data trustworthiness. This study proposes the DBG method to improve the data trustworthiness in the dynamic network. The system model was developed and applied with the SF attack to test the performance of the method. This study aimed to improve the data trustworthiness and security in IoT home appliances. The novelties of the proposed DBG method are as follows:

1. The proposed DBG method is based on bargaining techniques to improve the data trustworthiness of the IoT network. The bargaining technique in the DBG method applies disagreement measures to the nodes, thus helping to avoid malicious nodes in the IoT network.
2. The DBG search process is carried out in a distributed manner to enable node mobility to be effectively achieved in the dynamic network. Unlike existing methods, the DBG method is performed in a distributed manner and considers the node mobility, which helps to achieve an effective performance in a dynamic network.
3. Time Difference to Collision (TDTC) is applied in the method to measure the probability of the node collision, and the bargaining method helps to avoid malicious nodes in the IoT network. Avoiding the malicious nodes and collision of nodes in dynamic networks effectively improves the network packet delivery ratio.
4. The proposed DBG method and existing methods in IoT path selection are evaluated in the dynamic network and compared in terms of data trustworthiness, packet delivery ratio, throughput, and end-to-end delay.

This rest of this paper is organized as follows. Recent methods for data trustworthiness in the IoT are reviewed in Section 2. Section 3 presents the proposal, and introduces the system model and the DBG method. Next, Section 4 provides the experimental setup, and Section 5 presents the results and discussion. Finally, the conclusion of this research paper is provided in Section 6.

## 2. Literature Review

The Internet of Things (IoT) provides resilient data accessibility and different forms of system management. In addition, as introduced above, the IoT has a number of challenges, such as power management and data trustworthiness (DT). Various recent methods involved in IoT security, and their related background, are introduced in this section.

Abdalzaher and Muta [16] proposed a repeated game model to improve the data trustworthiness and clustered WSN in IoT networks. The developed model can reduce power consumption in retransmission due to a hardware failure in a cluster member. The collision in the delivery packets in cluster members is avoided based on the TDMA protocol. The proposed model differentiates between a malicious cluster member and a hardware failure in the cluster member. The developed method carries out isotropic or non-isotropic transmission from a cluster member to increase the data trustworthiness. The developed Pareto optimal method increases the data trustworthiness compared to the non-cooperative defense method in the IoT.

Li et al. [17] proposed Trustworthiness Enhanced Reliable Forwarding (TERF) for the IoT to avoid malicious nodes in the network. The dual trustworthiness framework consists of local and global trustworthiness in the network nodes. A weighted directed graph was applied to model the mobile IoT, and the TERF method was applied to measure the service degree and contact probability. This method measures the nodes’ familiarity and reduces the interference from malicious nodes, resulting in a significant improvement in the trustworthiness. The social similarity measures the association of mobile nodes with the personal centrality for the relative node importance and avoids malicious nodes. The TERF method performs the dot product of two-node trustworthiness to measure the social similarity, and employs the degree and local trustworthiness to measure the personal centrality. The TERF method improves stability and security, and reduces latency and the network cost. The model has low adaptability in dynamic networks due to the node familiarity measures.

Liu et al. [18] proposed a blockchain-based method called Tornado that consists of a corresponding algorithm and space-structured ledger in the IoT. Network scalability was increased based on the data structures and space-structured chain architecture. The collaborative proof-of-work was applied in this model for heterogeneous IoT devices in this analysis. The resource efficiency was improved in IoT devices based on Space Structured Greedy Heaviest-Observed Subtree (S2GHOST). Data trustworthiness was improved based on the dynamic weight assignment method in the S2GHOST model. The Tornado method improves the performance in terms of latency and resource efficiency in the IoT. The security of the model was less defensive in the heterogeneous IoT system due to the distributed manner used. Javanmardi et al. [19] proposed a security-aware task scheduler in IoT-fog networks called FUPE, based on fuzzy-based multi-objective Particle Swarm Optimization, to increase the security of protection. The FUPE method has higher security against DDoS attacks, and effectively detects the attack. The FUPE method combines the Software-Defined Network (SDN) and Fog Technology to protect against the DDoS attack. The FUPE method has a higher performance in terms of lower latency and security compared to the state-of-art methods. The model has poor convergence in the optimization process and can withstand a single attack.

Shijie and Yingfeng [20] applied the cross-entropy method and fuzzy Analytic Network Process to analyze the credit of Manufacturing Services (MS) tasks the in IoT. The blockchain method was applied to increase the data security in this model, and processing took place in the unified platform. The Service Scoring Mechanism (SSM) personalizes the service of credit evaluation and the smart service configuration model carries out the matching of demand and supply. The adaptability of the model was low and its security was lower in the dynamic network. The data trustworthiness of the model was affected by the fuzzy model of the sub-attributes. Roldán et al. [21] applied machine learning Complex Event Processing (CEP) to detect the different types of attacks in real-time IoT networks. Automatic code generation and attack prediction patterns based on the model-driven graphical tool were provided. The CEP model was applied to the healthcare IoT network to detect malicious nodes. Lee et al. [22] proposed a game theory method to quantify vulnerability and to increase the security in IoT networks. The developed model consists of three stages: game strategy, cost impact, and payoff calculation. The developed method has higher resilience than the existing methods in IoT networks.

Djedjig et al. [23] proposed a metric-based routing protocol that was applied to evaluate the secure routing topology. Game theory was applied to analyze the cooperation and MRTS was used to find the malicious node. The metric-based routing protocol method has higher efficiency in terms of node rank changes, energy consumption, and packet delivery ratio. Alzubi et al. [24] applied the Hermitian-Based Cryptosystem (HBC) to improve IoT security and to find malicious nodes. Kerckhofs’s desideratum was the main guidance to choose the Hermitian curve and generate encryption keys. The error connection shows that the Hermitian method has higher performance than the McEliece cryptosystem. Hayajneh et al. [25] applied the Software Definition Network (SDN) in the system model to improve the security of the IoT network. The developed method has the advantages of mitigating the Man-in-the-Middle attack, thereby increasing security. The results indicate the developed method shows higher resilience to the Man-in-the-Middle attack than existing methods.

Thirumalai et al. [26] presented a non-linear Diophantine equation to provide resilience against side-channel attacks such as timing attacks. The RSA and ESR were applied to provide three-stage encryption and two-stage decryption. The knapsack method was also applied in the developed method to increase the security in the IoT cloud. The key generation of the model shows that the developed method has higher resilience compared to the existing methods. Hu et al. [27] proposed a data trustworthiness enhanced Crowdsourcing Strategy (DTCS) method to increase the security in the IoT environment. The attribute relevancy and participant’s familiarity were assessed to select the node for the path and data transfer in the IoT cloud. The DTCS method increases the security of crowdsourcing and provides defense against behavior attacks and collision attacks. The DTCS method provides higher security compared to the existing methods of TSCM. Habib et al. [28] proposed modified multi-objective Particle Swarm Optimization (PSO) with the Levy flight method, which was applied for intrusion detection in the IoT network. A modification in the PSO method was applied to tackle the problem of feature selection in the network. The UCI repository data were used to test the performance of the optimization method, and the results show that the developed method has higher detection performance compared to existing methods.

Other researchers [16,17,18,19,20] achieved higher security in IoT networks using game theory, optimization, and attribute-based security models. However, the review of recent methods applied in the IoT shows that the existing approaches have the limitations of low data trustworthiness, low adaptability, and high latency.

## 3. Proposal

### 3.1. System Model

**Figure 1:**
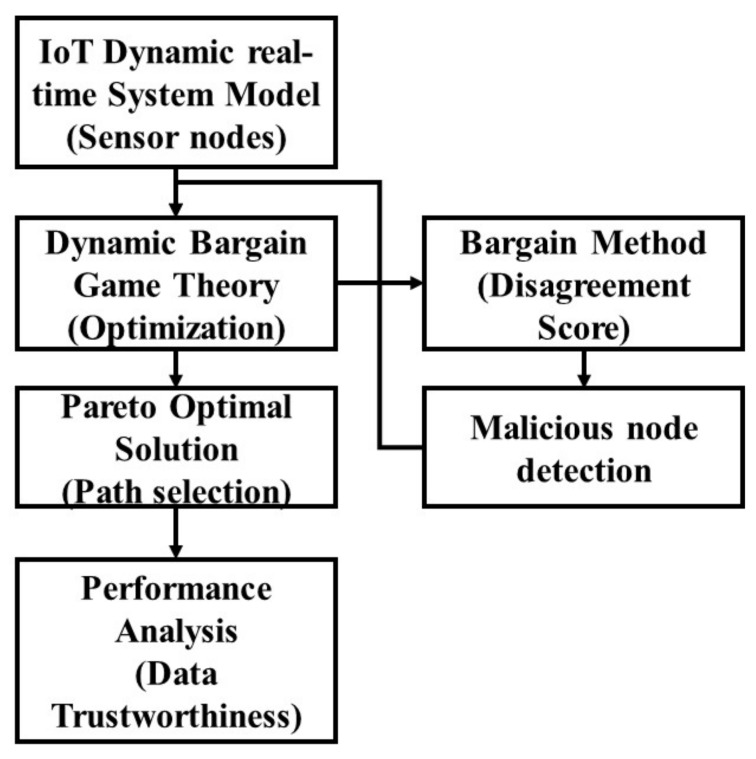
The block diagram of the Dynamic Bargain Game in the dynamic network.

The cluster members (CMs) set in WSN is used in this model, and the cardinality of the set N is represented as $\left|N\right|$, and defines the number of CMs, N, in the cluster. In the clustered WSN, the TDMA protocol is applied to manage the packet transmission and synchronization between the cluster head (CH) and CMs. The cluster head is responsible for collecting the information from cluster members to assign the task. The cluster head is selected randomly because changes in the cluster head do not have a large impact on the data transmission. The cluster head and cluster member are the sensor nodes in the network having mobility. Various sensors are used in real-time networks, as discussed previously, and these sensors are considered as nodes in the system model. Although the proposed DBG method is applicable in various MAC protocols, such as the OFDM-based protocol [16], the TDMA protocol is used in this method due to its power conservation features in the network. TDMA has been proven to provide a long network lifetime for the WSN-IoT devices [29,30,31]. DMAC is a MAC protocol based on the scheduled time of TDMA, and is efficient in the IoT-WSN because it avoids overhearing, prolongs the network life, and improves energy conservation [32]. The block diagram of the DBG method in the IoT-WSN model is shown in Figure 1. The TDMA protocol, which has adequate intensity, was applied to test the performance of the proposed DBG method [33,34,35,36,37,38].

The game theory-based approach is applied in this model and two action states are present for each game player. First, every *i*th CM needs to perform a drop (D), i.e., dropping packets of malicious action, or no drop (ND), i.e., not dropping packets. The drop is stopped, or the packets that are supposed to be sent to nodes are dropped, while no drop is transmitting the packets to respective nodes. The CM is performed with the D action to save battery power in the network. Second, the CH performs a no Beacon (NB) or Beacon (B) action for each CM based on the game theory decision. The benevolent CM is provided with permission and denoted as action B, i.e., it does not drop packets to send the observed data [32]. The sleep mode of CM is also activated based on the B permission and battery life is saved using a rest in packet transmission, and power recovery while recharging is available. If D is applied in the *i*th CM, then NB action is performed in the model. If an SF attack is performed on a network, a random hardware failure occurs in the real-time network due to CM performance. This hardware suffers from excessive packet transmission and dropping of packets that are considered in the proposed method. The proposed method effectively classifies the malicious CM due to the SF attack and the nodes that suffer from the hardware failure. The benevolent CM is applied with B based on the TDMA at the time of data transmission. A Selective Forwarding (SF) attack acts as a normal node and discards the critical information to the destination. This attack causes loss of important information and failure of smart devices due to this information loss. Traffic rate, duration, number of logins, and number of failed logins are important attributes used to detect the SF attack in game theory.

Node mobility is common in IoT networks, and is mostly a subset of an element in the network. The integration of mobile nodes, connectivity, and resource allocation are carried out in the model. The proposed method was developed to support the mobile nodes in IoT networks and improve the data trustworthiness. After CM behavior checking, the Beacon (B) action is distributed in the network. Initially, all CMs are considered to be benevolent and the cluster head (CH) action for all CMs is B. The CMs are calculated with the utility function of the *i*th CM ($U_{i}$), and the CH determines whether the CM has the right to be supported by the Beacon action based on the utility function. The TDMA performed on the *i*th CM determines if the CM is benevolent and can thus receive the Beacon. If the model finds that the *i*th CM is malicious, the Beacon is not sent to the *i*th CM. The continuous re-transmission of packets by the malicious node and acknowledgment are not received by the CH. If the battery power is weakened by the CM due to malicious behavior, then the CM is prone to die. All CMs are considered to be benevolent in the final round; the total number of rounds in the given period is denoted as $N_{rd}$ and hardware failure is not considered. A star topology is applied for the communication between CMs and the CH, and a dedicated communication link between them is used to facilitate the checking procedure of the received packets for every *i*th CM. A star topology is also applied to maintain stable communication between the CH and CMs in the dynamic network. A stark topology has the capability to change the CH if one of the CH fails, thus improving the stability in the dynamic network. CM communication to other clusters is through the CH, and a conventional topology in the real-time network is applied to determine the benevolent CMs that do not drop. Four scenarios were considered relating to the CH action ($A_{i}$ CH) and the *i*th CM action ($A_{i}$). Four representations of three scenarios denote the one-shot games in which the same action is performed by the CM and the CH regardless of other players’ actions. The rational interaction between the *i*th CM and the CH is the last scenario to decide the reward and punishment to act as a defense mechanism, and is carried out by every CM action. The behavior of all of the malicious rational CMs becomes benevolent based on this method. The four action states of the two players (i.e., the *i*th CM and CH) are denoted based on following processes:

$$A_{CH}^{i}=NB\;\mathrm{and}\;A_{i}=D\;A_{CH}^{i}=NB\;\mathrm{and}\;A_{i}=ND\;A_{CH}^{i}=B\;\mathrm{and}\;A_{i}=D\;A_{CH}^{i}=B\;\mathrm{and}\;A_{i}=ND$$

The system model performance is explained as follows:

1. The game theory method is used to decide to perform drop or no drop;
2. Every ith CM must perform a drop or no drop action based on the game theory decision;
3. Drop is performed to send the sensor data to the destination;
4. No drop equates to a denial of transmission of the data;
5. No drop is performed for two reasons:
  a. Game theory labels the node as malicious;
  b. The system performs no drop to save power (sleep mode). In this case, the drop is activated once the node is recharged;
6. CH has two action states: Beacon or No Beacon. This action is decided by game theory.
  a. Beacon provides permission for no drop, to go into sleep mode, and re-activation from the sleep mode;
  b. No Beacon denies data transmission for the node.
7. Beacon action is performed after checking the behavior of the CMs;
8. The CH monitors the communication between the CMs;
9. The game theory method is performed for CH decisions.

#### Special Cases

There is a chance that the malicious node and hardware failure node will suffer from the no drop. The proposed game theory model is able to differentiate between nodes experiencing a hardware failure and those subject to an SF attack.

### 3.2. Dynamic Bargain Game Method

Game theory is an approach for decision making based on several players characterized by various conflicts of interest and mutually interdependent situations. Co-operation and interaction are the important processes in game theory, which is considered a rational method to solve conflict based on the node interaction. The game theory tool is used to obtain the negotiation process, which is transformed from intersection resolution. The CH consists of data related to the CMs and communication between the CMs. Game theory uses monitoring to determine if the node is malicious. The major objective of game theory is to analyze the node data from the CH monitoring data to determine if the node is malicious. Game theory selects the possible path based on the objectives to provide a suitable path to effectively transfer the data. If nodes are represented as players in game theory, more possible paths are selected in the method and irrelevant paths are also considered for the objective. The consideration of more possible and irrelevant paths results in low efficiency and high computational complexity. The typical scenario of game theory is described as follows.

The players: The players are regarded as entities that influence the game outcome and are denoted as $G=\left\{V_{1},\;V_{2},\;V_{3}\right\}$.

The strategies: The game strategy set comprises a player’s sequence of actions that are undertaken to complete a plan. This strategy is used to decide if an action is necessary for the particular step, and the set of strategies is denoted as $S=\left\{Accelerate,\;Uniform,\;Decelerate\right\}$. The strategy denotes the states of the node, which are increase speed, maintain speed, and decrease speed.

The payoff: At the end of the game, the payoff is applied to benefit the players who followed their strategy. Each player has a payoff set of $P_{m}=\left\{S_{1},\;S_{2},\;S_{3}\right\},\;m=1,\;2,\;3$.

In this stage, the acceleration element is classified into three categories and strategy is varied in the range of $\left[a_{min},\;a_{max}\right]$, which is different from the deceleration and acceleration of the fixed value in a static game.

#### 3.2.1. Payoff Design

This method focuses on three important aspects (three players) of the network, namely, efficiency, safety, and interaction. A safe intersection and more efficiency between the nodes are considered to be objectives in this method. The conflicts of place and time are predicted based on the neighbors’ shared states and the unsignalized intersection is followed in this method. The co-operation method is applied to increase the network traffic in the shared states, and velocity changes influence the efficiency. Control strategies in the node aspects are limited to the node’s execution capacity (velocity of the node).

The payoff is sorted into two levels based on different aspects by considering the safety and efficiency intersection level. The execution capability is considered at the node level and the payoff is given as follows in Equations (1) and (2).

(1) $$f_{p,m}^{i}=\alpha_{p,m}T_{m}^{i}+\beta_{p,m}\Delta T_{m,n}^{i}-\gamma_{p,m}\Delta v_{m}^{i}+\delta_{p,m}{\Delta \alpha }_{m}^{i},\;$$

(2) $$m,n\in \left\{1,2,3,m\neq n\right\},i\in N$$

where the pair of nodes, node ID, priority are denoted as mn, m, and p subscripts and the *i*th iteration is denoted in superscript. The node weight elements are denoted as $\alpha_{p,m}$, $\beta_{p,m}$, $\gamma_{p,m}$, and $\delta_{p,m}$, respectively. Matrices $T_{m}^{i}={\left[T_{1}^{i},T_{2}^{i},T_{3}^{i}\;\right]}^{T}$ and $\Delta T_{mn}^{i}={\left[\Delta T_{12}^{i},\;\Delta T_{13}^{i},\;\Delta T_{23}^{i}\right]}^{T}$ denote the weights corresponding to $\alpha_{p,m}=\left[\alpha_{p,m1},\;\alpha_{p,m2},\;\alpha_{p,m3}\right]$ and $\beta_{p,m}=\left[\beta_{p,m12},\;\beta_{p,m13},\;\beta_{p,m23}\right]$, respectively. The matrix transpose operation is denoted using the superscript “*T*” and weights are satisfied using the following condition in Equation (3).

(3) $$W\cdot \alpha_{p,m}^{T}+W\cdot \beta_{p,mn}^{T}+\gamma_{p,m}+\delta_{p,m}=1$$

where $W=\left[1,1,1\right]$, and various weights combinations are denoted based on the interaction degree and various driving characteristics.

The payoff of each level in each related element is given as follows.

Intersection level in the payoff: The intersection level in the payoff focuses on the nodes that are described as safe and efficient.

The negotiation game of crossing efficiency is the key aspect. The TTC is applied for each node with parameters of $p_{m}$, $v_{m}$, and $a_{m}$, as given in Equation (4).

(4) $$\left\{\begin{array}{c} T_{m}^{i}=\sqrt{{\left(\frac{v_{m}^{i}}{a_{m}^{i}}\right)}^{2}+\left(\frac{2p_{m}^{i}}{a_{m}^{i}}\right)}-\left(\frac{v_{m}^{i}}{a_{m}^{i}}\right),m=1,2,3,i\in N,\;\\ v_{min}\leq v_{m}^{i}\leq v_{max} \end{array}\right.$$

The constraint is set for the minimum and maximum velocity, and network traffic rules are applied in the method. The acceleration, velocity, and collision point position are denoted as $p_{m}^{i}$, $v_{m}^{i}$, and $a_{m}^{i}$ in a certain iteration loop, respectively.

The basic principle of cooperative intersections is the safe transmission of data. Each node safety pair is denoted as the Time Difference to Collision (TDTC), as given in Equation (5).

(5) $$\Delta T_{mn}^{i}=\left|T_{m}^{i}-T_{n}^{i}\right|,m,n\in \left\{1,2,3,n\neq m\right\},i\in N$$

Each node’s safety is defined as the TDTC in a certain loop, which indicates each node with the appropriate time to transfer the data. Equation (5) denotes the two nodes’ time difference to consecutively pass the data while ensuring safety. The interaction crossing efficiency is denoted in Equation (6).

Equation (6) is defined based on the third term in Equation (1).

(6) $$\Delta v_{m}^{i}=v_{m}^{i+1}-v_{m}^{i},m=1,2,3,i\in N$$

The velocity change and initial velocity affect the network traffic efficiency. A positive value indicates an improvement in the node speed, and node slowdown is denoted by a negative value. An improvement in network traffic efficiency is indicated by a negative sign for the node velocity.

Node Payoff: The acceleration and deceleration of nodes affect the efficiency and safety of the nodes. The acceleration and deceleration change is denoted in Equation (7).

(7) $$\Delta a_{m}^{i}=\left|a_{m}^{i}-a_{m}^{i-1}\right|,m=1,2,3,i\in N$$

Normalization: Different parts with different dimensions are considered based on a zero-mean normalization, as adopted in Equation (8).

(8) $$y^{*}=\frac{y-\mu_{y}}{\sigma_{y}}$$

where the original input and the normalized input are denoted as $y,\;y^{*}$, $\mu_{y}$ denotes the y expectation, and $\sigma_{y}$ is the standard deviation of y.

#### 3.2.2. Pareto Optimal Set

A satisfactory solution of the Pareto optimal solution is introduced in this method. The best payoff for each node is not ensured by minimizing the global payoff. The genetic algorithm is applied to select the minimum global payoff in the search for the Pareto optimal solution. The optimal solution is regarded as a fitness function, as given in Equation (9).

(9) $$F_{p}^{i}=\omega_{p,1}^{i}\cdot f_{p,1}^{i}+\omega_{p,2}^{i}\cdot f_{p,2}^{i}+\omega_{p,3}^{i}\cdot f_{p,3}^{i}$$

where payoff functions $f_{p,1}^{i}$, $f_{p,2}^{i}$, and $f_{p,3}^{i}$ considered in the $i^{th}$ generation are provided in Equation (1), and are measured using the strategy set $\left\{a_{1}^{i},\;a_{2}^{i},\;a_{3}^{i}\right\}$. The coefficient weights are denoted as $\omega_{p,1}^{i},\;\omega_{p,2}^{i}$, and $\omega_{p,3}^{i}$. Each generation i is constantly applied in $a_{min}=-2\;m/s^{2}$, $a_{max}=2\;m/s^{2}$. The global optimal strategy set is applied after the genetic algorithm standard procedure.

#### 3.2.3. Bargaining Game

A bargaining game is applied to improve the mutual interaction between the nodes using disagreement point $d\left(k\right)$ and decision space Υ, which is defined as $\left\{\left(\Upsilon ,\;d\left(k\right)\right)\right\}$.

The general game is used to formulate the bargaining and to search for the Pareto optimal set, and each node-weighted global payoff is minimized to formulate the game problem. The game problem is defined in Equation (10).

(10) $$\underset{u\left(k\right)}{\min}\sum_{m=1}^{M}\omega_{m}\phi_{m}\left(u\left(k\right)\right)$$

$$S.t.\;u_{m}\left(k\right)\in u_{m},m=1,2,3$$

where each node weight coefficient payoff is M=3 and $\omega_{m}$.

The fitness function is applied to the disagreement point to develop the bargaining game based on cooperative game theory. The disagreement point $d_{m}\left(k\right)$ at time step k is denoted as $d_{m}\left(k\right)=\varphi_{m}\left(u^{p}\left(k\right)\right)$, and $u^{p}\left(k\right)$ is obtained to solve the problem, as shown in Equation (11).

(11) $$\underset{u_{m}\left(k\right)}{\min}\underset{u_{-m}\left(k\right)}{\max}\phi_{m}\left(u\left(k\right)\right)$$

$$S.t.\;u_{m}\left(k\right)\in u_{m},m=1,2,3$$

$$u_{-m}\left(k\right)\in u_{-m},m=1,2,3$$

where the node m strategy set is denoted as $u_{-m}\left(k\right)$, the worst-case node is denoted as G, and the best benefit is denoted as $d_{m}\left(k\right)$, which is used to measure the worst case.

The disagreement point of the bargaining game based on the Nash solution is given in Equation (12).

(12) $$\underset{u\left(k\right)}{\max}\overset{M}{\underset{m=1}{\prod }}{\left[d_{m}\left(k\right)-\phi_{m}\left(u\left(k\right)\right)\right]}^{\omega_{m}}$$

$$S.t.\;d_{m}\left(k\right)>\phi_{m}\left(u\left(k\right)\right),\;m=1,2,3$$

$$u_{m}\left(k\right)\in u_{m},m=1,2,3$$

The maximization problem is rewritten equivalently in Equation (13).

(13) $$\underset{u\left(k\right)}{\max}\overset{M}{\underset{m=1}{\sum }}\omega_{m}\log\left[d_{m}\left(k\right)-\phi_{m}\left(u\left(k\right)\right)\right]$$

$$S.t.\;d_{m}\left(k\right)>\phi_{m}\left(u\left(k\right)\right),m=1,2,3$$

$$u_{m}\left(k\right)\in u_{m},m=1,2,3$$

Problem (13) is solved in a distributed manner using the feasible-cooperation method. The application of the greedy method in a strategy that focuses on the local payoff provides more benefit in the cooperation manner; thus, the greedy strategy was applied in the current iteration.

## 4. Experimental Setup

This study applied the Dynamic Bargain Game method in the IoT network to improve the data trustworthiness and performance. The DBG method improves the efficiency of data transfer and maintains the security of the method. This section provides the details of the network parameters, metrics, and system requirements of the proposed DBG method. The parameters of the network are given in Table 1. The given parameter settings are common for this network and are applicable to dynamic nodes in the network.

Metrics: The DT calculated by the CH is defined as the average utility function of all CMs during the given time period, as shown in Equation (14).

(14) $$DT=\frac{\sum_{rd=1}^{N_{rd}}\sum_{i=1}^{\left|N\right|}U_{i}^{rd}}{N_{rd}}$$

System Requirement: The proposed method was implemented on a system consisting of an Intel i9 processor, 128 GB of RAM, a 22 GB hard disk, and a Windows 10 64-bit OS. The network simulator 2.35 (NS-2) was used to simulate the network model and test the proposed DBG method.

Dataset: The input dataset consists of 11 columns and 148 rows related to sensor information. The rows denote the number of smart rooms and the columns denote the attributes of the collected information. The column attributes consist of thermistor sensor, CMOS sensor, humidity sensor, PIR sensor, total size of the data, traffic rate, duration, urgency, server error, number of logins, and number of failed logins. Sensor data denotes the value of the collected information; total size of the data denotes the data size of all sensors; traffic rate is considered to be an important attribute for security; duration denotes the connection time of the sensors; server error denotes the package loss in transmission; number of logins is the number of users present in the cloud environment; and number of failed logins is an important attribute for security. Because users can login to the cloud environment to check the sensor data of their specific ids, the number of logins and number of failed logins are measured.

## 5. Results and Discussion

This study applied the DBG method to perform transmission with increased data trustworthiness and improved security. The DBG method is applied in the system model to identify malicious nodes and differentiate nodes experiencing a hardware failure from malicious nodes. The bargaining concept is applied using game theory to increase the interaction of the nodes, and the Pareto optimal method is applied to identify suitable nodes to transfer the data with higher data trustworthiness. Various metrics were assessed, such as data trustworthiness, packet delivery ratio, throughput, and end-to-end delay.

The DBG method and existing methods were applied in the simulated system model to evaluate the data trustworthiness, as shown in Table 2. The data trustworthiness was measured based on the selection of trusted nodes to transfer the data to the destination. The DBG method applies the Pareto optimal solution to determine the fitness values for the nodes to transfer the data, and the bargaining method applies the disagreement point to avoid malicious nodes in the path. The cooperation process of the DBG is improved by performing the search in a distributed manner in the network. The Pareto optimal solution in the DBG method effectively handles the tradeoff between safety and efficiency. These advantages in the DBG method improve the data trustworthiness of the network compared to existing models. The fuzzy cross entropy [20] model has the second higher performance in terms of data trustworthiness. The Pareto optimal [16] solution provides a considerable increase in performance by improving the data trustworthiness. The existing models have the limitation of low adaptability in dynamic networks and lower efficiency in choosing the node for the data transmission.

**Figure 2:**
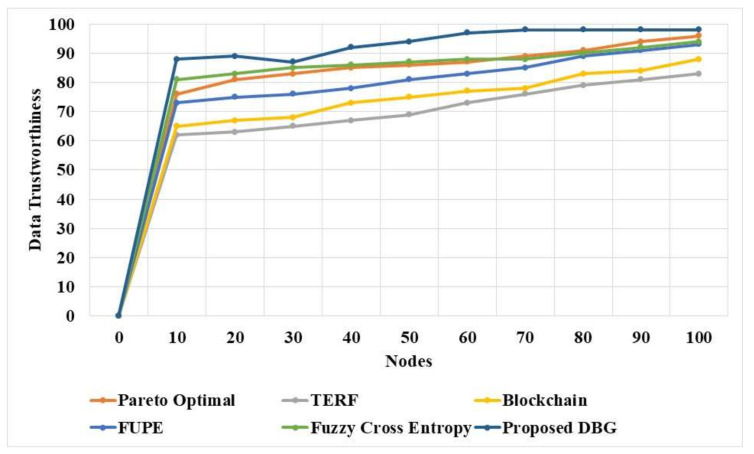
Data trustworthiness of the DBG method.

The DBG and existing methods were applied in the simulated network to test the node selected for data trustworthiness, and are compared in Figure 2. The DBG method has higher data trustworthiness than existing methods due to the Pareto optimal solution for the security, and the bargaining method for eliminating the malicious nodes in the path. The fuzzy cross entropy model [20] achieved the second highest performance in terms of data trustworthiness, and the Pareto optimal [16] solution demonstrated a strong performance. FUPE [19] based on the multi-objective PSO method showed a weaker performance due to poor convergence. The fuzzy cross entropy [20] and Pareto optimal [16] methods have the limitation of lower adaptability in the dynamic network. The DBG method shows higher data trustworthiness for more nodes than existing methods. The DBG method performs the search process in a distributed manner and is therefore an effective solution in dynamic networks.

**Figure 3:**
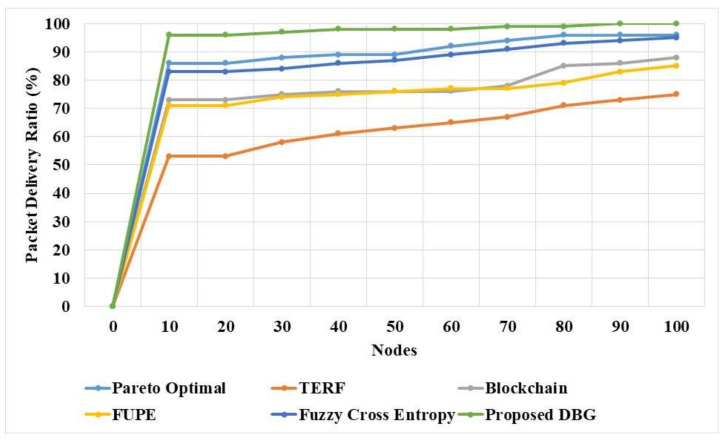
Packet delivery ratio of the DBG method.

The DBG and existing methods were evaluated based on the packet delivery ratio in the dynamic network environment, and the comparison is provided in Figure 3 and Table 3. The packet delivery ratio was usually affected by the collision of the nodes in the data transmission and the attack of the malicious nodes. The bargaining method applied the disagreement in the nodes, based on the node activity, to eliminate the malicious nodes. The TDTC approach was applied in the DBG method to analyze the possible collisions in the search process, and thus avoid the collisions. The existing methods failed to eliminate the collision possibilities and the performance for the detection of malicious nodes was weaker. The packet delivery ratio shows that the DBG method has higher security than that of the existing methods.

**Figure 4:**
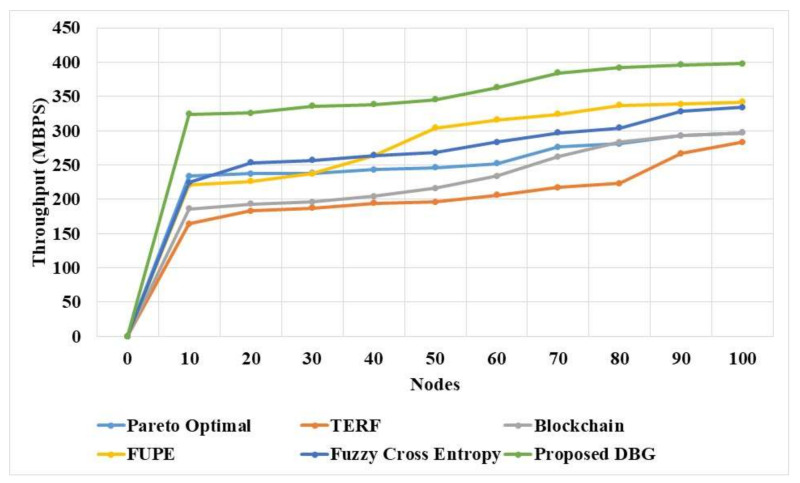
Throughput of the DBG method in a dynamic network.

The DBG and existing methods were evaluated based in throughput in a dynamic network to analyze the capacity of the method to transfer the data, as shown in Figure 4 and Table 4. The DBG method attained a higher throughput in the dynamic method than the existing methods due to the efficiency of its Pareto optimal solution. The DBG method considers the data trustworthiness and efficiency in the Pareto optimal solution when forwarding the data to the nodes. The cooperation in the DBG method is involved in the selection of the optimal path, thus increasing the data transmission capacity, and this process increases the throughput of the DBG method. The Pareto optimal [16] method selects the path based on the sole objective of data trustworthiness, and this affects the efficiency of the method. The fuzzy cross entropy [20] method has lower adaptability in the dynamic network, which affects the efficiency of the method.

**Figure 5:**
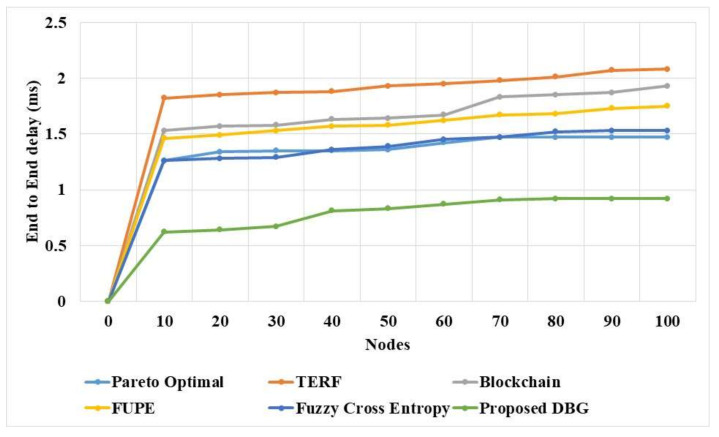
End-to-end delay of the proposed DBG method.

The end-to-end delay of the DBG method was compared with that of existing methods in a dynamic environment, as shown in Figure 5 and Table 5. The DBG method has a lower end-to-end delay than existing methods in the dynamic network. The DBG method searches for the solution in a distributed manner, and this helps to find the effective solution for the data transfer. The fuzzy cross entropy method [20] and Pareto optimal [16] solution have lower adaptability in the dynamic network. FUPE has poor convergence in the multi-objective PSO method for the node selection in the network.

The network utilization of the proposed DBG and existing methods in a dynamic environment is presented in Table 6. These results show that the proposed DBG method has lower network utilization compared to the existing methods. The DBG method has a Pareto optimal solution, which improves the security and eliminates the malicious nodes that reduce the network utility of the model. The fuzzy cross entropy [20] and Pareto optimal [16] methods have lower performance due to their low adaptability in the model. The FUPE [19] method has poor convergence, which affects the performance of the model.

**Figure 6:**
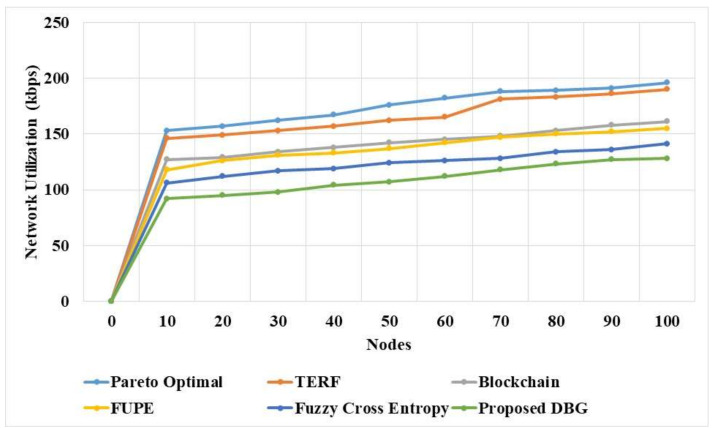
Network utilization of DBG and existing methods.

The network utilization of the proposed DBG and existing methods in a dynamic network is compared in Figure 6. The DBG method has the advantage of performing the search process in the distributed manner that improves the efficiency of the network. The Pareto optimal [16] and fuzzy cross entropy [20] methods have lower adaptability, which increases the network utilization. The FUPE [19] method has poor convergence in the optimization process, which increases the network utilization.

**Figure 7:**
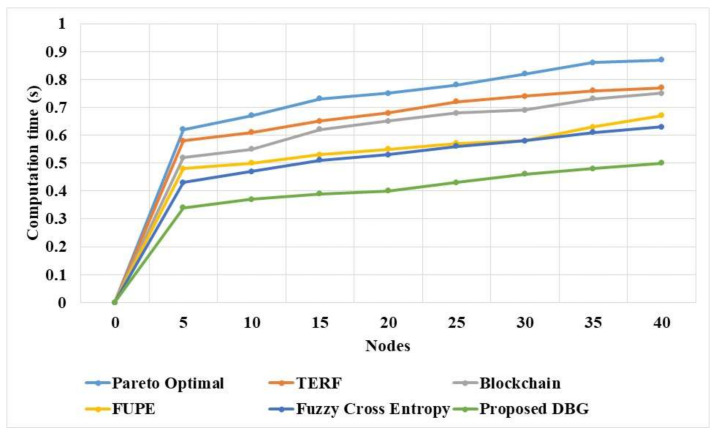
Computation time of the DBG and existing methods for various CMs.

The computational time of the DBG and existing methods for various CMs is shown in Table 7 and Figure 7. The DBG method performs the search process in a distributed manner and eliminates the malicious nodes. This reduces the computation time of the data transfer of malicious nodes and enables the process to be performed in a parallel manner. The Pareto optimal solution in the DBG method improves the security and reduces the computation time. The FUPE [19] method requires an optimization process for node selection and has poor convergence, which improves the computation time of the method. The Pareto optimal [16], TERF [17], and fuzzy cross entropy [20] methods have low adaptability, which increases the computation time for node selection and identification of malicious nodes. This shows that the proposed DBG method has higher performance compared to the existing methods in terms of security and efficiency.

**Figure 8:**
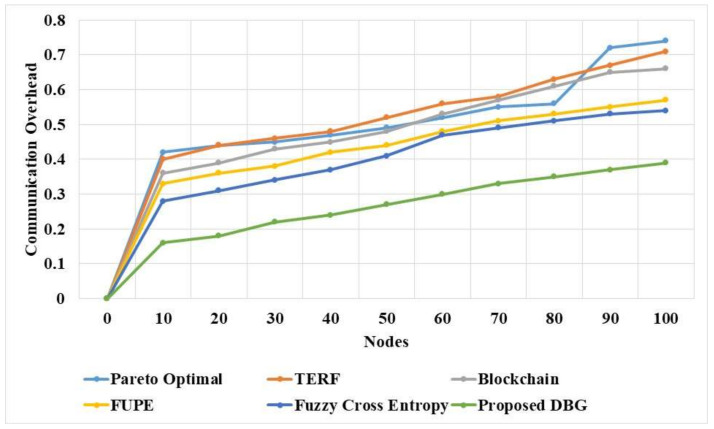
Communication overhead of the DBG and existing methods.

The communication overhead of the DBG and existing methods for various nodes in a dynamic network is compared in Table 8 and Figure 8. The DBG method performs the search process in a distributed manner to select the node, and transmits the data after node selection. The Pareto optimal approach in the DBG method helps to effectively eliminate the malicious nodes in the network. The DBG method has less communication overhead due to the elimination of malicious nodes and the selection of nodes in a distributed manner. The FUPE [19] method has the limitation of poor convergence, which affects the performance of the model. The Pareto optimal [16], TERF [17], and fuzzy cross entropy [20] methods have lower adaptability in the network.

**Figure 9:**
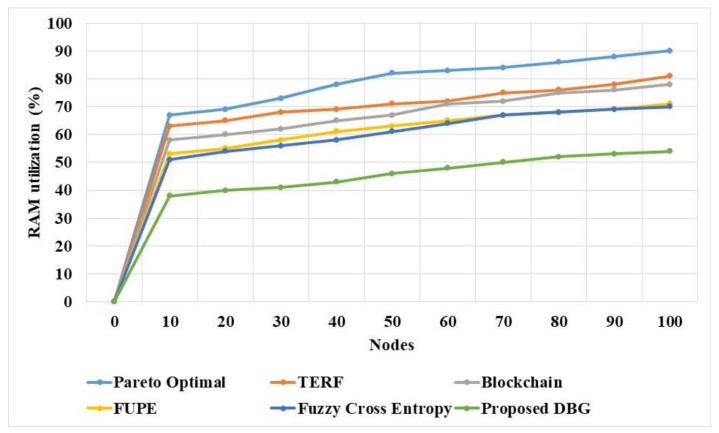
RAM utilization of the DBG and existing methods.

The RAM utilization of the DBG and existing methods was measured for various nodes, and is compared in Table 9 and Figure 9. These results show that the RAM utilization of the proposed DBG method is low compared to that of existing methods. The proposed DBG method discards the data used for the search process because it is not required to be processed in the dynamic network. The DBG method stores the data related to the nodes and malicious nodes for the node selection. The existing methods generate more data to identify the suitable nodes for transmission in the optimization process and this tends to improve the memory requirement.

The proposed method is applicable to smoke sensors (infra-red LED sensors) for fire detection, camera sensors (CMOS sensors) for security alarms, temperature sensors (electronic thermistor sensors) for smart thermostats, moisture detection sensors (humidity sensors) for leak/moisture detection, and Passive Infrared (PIR) in motion sensors. The application of the proposed method to the network instead of individual sensors reduces the cost of the network.

## 6. Conclusions

The existing methods used to select nodes for data transmission have low efficiency in dynamic networks and exhibit a poor performance for malicious node detection. Smart home devices are used to control various home appliances and require high security to protect the privacy of the users in real-time networks. A security measure is required for IoT-based smart home systems to provide high data trustworthiness and low end-to-end delays. This research proposed the DBG method for the selection of the nodes for data transmission, to increase the data trustworthiness and efficiency of the network. The data trustworthiness and efficiency were applied as the objective function in the Pareto optimal solution for node selection. The bargaining technique in the DBG method assigns the disagreement to the node, which eliminates the malicious nodes in the node selection process. The DBG method of the TDTC process considers the possible collision of a node in the network and selects the node to eliminate the collision. The DBG method searches in a distributed manner, thus providing a better solution for dynamic networks. The packet delivery ratio shows that the DBG method has higher resilience than the existing methods. The developed DBG method improved the data trustworthiness and effectiveness in terms of throughput, end-to-end delay, and resource utilization. The communication overhead was reduced up to 27% and RAM utilization was reduced up to 16%. The throughput and end-to-end delay show the DBG method has higher efficiency than the existing methods. The DBG method has data trustworthiness of 98%, compared to the fuzzy cross entropy method, which has data trustworthiness of 94%. In future work, this method will be applied to the attack detection model to provide security against various attacks.

## Figures and Tables

**Table 1:** The parameter settings of the network model.

Parameters	Value
Total number of CM	1 and 2
Offset Start-up time	580 µs
Transmission Data Rate	250 kb/s
Battery voltage of sensor devices	3 Volts
Transmission energy per bit	50 nJ
Distance between the *i*th CM and CM	125 m
Transmission power level	31
Total number of packages per second	11

**Table 2:** Data trustworthiness of the DBG method.

Nodes	Pareto Optimal [16]	TERF [17]	Blockchain [18]	FUPE [19]	Fuzzy Cross Entropy [20]	DBG
0	0	0	0	0	0	0
10	76	62	65	73	81	88
20	81	63	67	75	83	89
30	83	65	68	76	85	87
40	85	67	73	78	86	92
50	86	69	75	81	87	94
60	87	73	77	83	88	97
70	89	76	78	85	88	98
80	91	79	83	89	90	98
90	94	81	84	91	92	98
100	96	83	88	93	94	98

**Table 3:** The packet delivery ratio of the DBG method.

Nodes	Pareto Optimal [16]	TERF [17]	Blockchain [18]	FUPE [19]	Fuzzy Cross Entropy [20]	DBG
0	0	0	0	0	0	0
10	86	53	73	71	83	96
20	86	53	73	71	83	96
30	88	58	75	74	84	97
40	89	61	76	75	86	98
50	89	63	76	76	87	98
60	92	65	76	77	89	98
70	94	67	78	77	91	99
80	96	71	85	79	93	99
90	96	73	86	83	94	100
100	96	75	88	85	95	100

**Table 4:** Throughput of the proposed DBG method.

Nodes	Pareto Optimal [16]	TERF [17]	Blockchain [18]	FUPE [19]	Fuzzy Cross Entropy [20]	DBG
0	0	0	0	0	0	0
10	234	164	186	221	224	324
20	237	183	193	226	253	326
30	238	187	196	237	257	336
40	243	194	204	264	264	338
50	246	196	216	304	268	345
60	252	206	234	316	283	363
70	276	217	262	324	297	384
80	281	223	283	337	304	392
90	293	267	293	339	328	396
100	297	283	297	342	334	398

**Table 5:** The end-to-end delay of the proposed DBG method.

Nodes	Pareto Optimal [16]	TERF [17]	Blockchain [18]	FUPE [19]	Fuzzy Cross Entropy [20]	DBG
0	0	0	0	0	0	0
10	1.26	1.82	1.53	1.46	1.26	0.62
20	1.34	1.85	1.57	1.49	1.28	0.64
30	1.35	1.87	1.58	1.53	1.29	0.67
40	1.35	1.88	1.63	1.57	1.36	0.81
50	1.36	1.93	1.64	1.58	1.39	0.83
60	1.42	1.95	1.67	1.62	1.45	0.87
70	1.47	1.98	1.83	1.67	1.47	0.91
80	1.47	2.01	1.85	1.68	1.52	0.92
90	1.47	2.07	1.87	1.73	1.53	0.92
100	1.47	2.08	1.93	1.75	1.53	0.92

**Table 6:** Network utilization of the DBG method.

Nodes	Pareto Optimal [16] (kbps)	TERF [17] (kbps)	Blockchain [18] (kbps)	FUPE [19] (kbps)	Fuzzy Cross Entropy [20] (kbps)	DBG (kbps)
0	0	0	0	0	0	0
10	153	146	127	118	106	92
20	157	149	129	126	112	95
30	162	153	134	131	117	98
40	167	157	138	133	119	104
50	176	162	142	137	124	107
60	182	165	145	142	126	112
70	188	181	148	147	128	118
80	189	183	153	150	134	123
90	191	186	158	152	136	127
100	196	190	161	155	141	128

**Table 7:** Computation time for various CMs.

Nodes	Pareto Optimal [16]	TERF [17]	Blockchain [18]	FUPE [19]	Fuzzy Cross Entropy [20]	DBG
0	0	0	0	0	0	0
5	0.62	0.58	0.52	0.48	0.43	0.34
10	0.67	0.61	0.55	0.5	0.47	0.37
15	0.73	0.65	0.62	0.53	0.51	0.39
20	0.75	0.68	0.65	0.55	0.53	0.4
25	0.78	0.72	0.68	0.57	0.56	0.43
30	0.82	0.74	0.69	0.58	0.58	0.46
35	0.86	0.76	0.73	0.63	0.61	0.48
40	0.87	0.77	0.75	0.67	0.63	0.5

**Table 8:** Communication overhead of the DBG method.

Nodes	Pareto Optimal [16]	TERF [17]	Blockchain [18]	FUPE [19]	Fuzzy Cross Entropy [20]	DBG
0	0	0	0	0	0	0
10	0.42	0.4	0.36	0.33	0.28	0.16
20	0.44	0.44	0.39	0.36	0.31	0.18
30	0.45	0.46	0.43	0.38	0.34	0.22
40	0.47	0.48	0.45	0.42	0.37	0.24
50	0.49	0.52	0.48	0.44	0.41	0.27
60	0.52	0.56	0.53	0.48	0.47	0.3
70	0.55	0.58	0.57	0.51	0.49	0.33
80	0.56	0.63	0.61	0.53	0.51	0.35
90	0.72	0.67	0.65	0.55	0.53	0.37
100	0.74	0.71	0.66	0.57	0.54	0.39

**Table 9:** RAM utilization of the DBG method.

Nodes	Pareto Optimal [16] (%)	TERF [17] (%)	Blockchain [18] (%)	FUPE [19] (%)	Fuzzy Cross Entropy [20] (%)	DBG (%)
0	0	0	0	0	0	0
10	67	63	58	53	51	38
20	69	65	60	55	54	40
30	73	68	62	58	56	41
40	78	69	65	61	58	43
50	82	71	67	63	61	46
60	83	72	71	65	64	48
70	84	75	72	67	67	50
80	86	76	75	68	68	52
90	88	78	76	69	69	53
100	90	81	78	71	70	54
